# Development of a simple standardized scoring system for assessing large vessel vasculitis by 18F-FDG PET-CT and differentiation from atherosclerosis

**DOI:** 10.1007/s00259-023-06220-5

**Published:** 2023-04-28

**Authors:** Y. A. A. Bacour, M. P. van Kanten, F. Smit, E. F. I. Comans, M. Akarriou, H. C. W. de Vet, A. E. Voskuyl, C. J. van der Laken, Y. M. Smulders

**Affiliations:** 1 Department of Internal Medicine, Amsterdam University Medical Center, Amsterdam 1007MB, PO Box 7057, Amsterdam, The Netherlands; 2 Department of Radiology and Nuclear Medicine, Alrijne Hospital, Simon, Smitweg 1, 2353GA Leiderdorp, The Netherlands; 3 Department of Radiology and Nuclear Medicine, Amsterdam University Medical Center, PO Box 7057, Amsterdam 1007MB, Amsterdam, The Netherlands; 4 Department of Radiology and Nuclear Medicine, Spaarne Hospital, PO Box 770, Hoofddorp, 2130AT The Netherlands; 5 Department of Epidemiology and Data Science, Amsterdam Public Health Research Institute, Amsterdam University Medical Center, VrijeUniversiteit Amsterdam, Amsterdam 1007MB, PO Box 7057, Amsterdam, The Netherlands; 6 Amsterdam UMC-Location VUmc, Amsterdam Rheumatology and Immunology Center (ARC), Amsterdam 1007MB, PO Box 7057, Amsterdam, The Netherlands

**Keywords:** Vasculitis, Atherosclerosis, 18-Fluorodeoxyglucose positron emission tomography, Computed tomography, Diagnosis

## Abstract

**Purpose:**

This study is to develop a structured approach to distinguishing large-artery vasculitis from atherosclerosis using 18-fluorodeoxyglucose positron emission tomography combined with low-dose computed tomography (FDG PET/CT).

**Methods:**

FDG PET/CT images of 60 patients were evaluated, 30 having biopsy-proven giant cell arteritis (GCA; the most common form of large-artery vasculitis), and 30 with severe atherosclerosis. Images were evaluated by 12 nuclear medicine physicians using 5 criteria: FDG uptake pattern (intensity, distribution, circularity), the degree of calcification, and co-localization of calcifications with FDG-uptake. Criteria that passed agreement, and reliability tests were subsequently analysed for accuracy using receiver operator curve (ROC) analyses. Criteria that showed discriminative ability were then combined in a multi-component scoring system. Both initial and final ‘gestalt’ conclusion were also reported by observers before and after detailed examination of the images.

**Results:**

Agreement and reliability analyses disqualified 3 of the 5 criteria, leaving only FDG uptake intensity compared to liver uptake and arterial wall calcification for potential use in a scoring system. ROC analysis showed an area under the curve (AUC) of 0.90 (95%CI 0.87–0.92) for FDG uptake intensity. Degree of calcification showed poor discriminative ability on its own (AUC of 0.62; 95%CI 0.58–0.66). When combining presence of calcification with FDG uptake intensity into a 6-tiered scoring system, the AUC remained similar at 0.91 (95%CI 0.88–0.93). After exclusion of cases with arterial prostheses, the AUC increased to 0.93 (95%CI 0.91–0.95). The accuracy of the ‘gestalt’ conclusion was initially 89% (95%CI 86–91%) and increased to 93% (95%CI 91–95%) after detailed image examination.

**Conclusion:**

Standardised assessment of arterial wall FDG uptake intensity, preferably combined with assessment of arterial calcifications into a scoring method, enables accurate, but not perfect, distinction between large artery vasculitis and atherosclerosis.

**Supplementary Information:**

The online version contains supplementary material available at 10.1007/s00259-023-06220-5.

## Introduction

Giant cell arteritis (GCA) is the most prevalent form of large-artery vasculitis in elderly patients. Delay in treatment may cause severe complications, such as aneurysm, dissection and stenosis. Early diagnosis and adequate treatment on the other hand can lead to complete remission of the disease [1].

The current reference test for GCA is biopsy of the temporal artery. Biopsy, however, is invasive and the temporal artery is not always affected in GCA [2, 3]. More recently, 18-fluorodeoxyglucose positron emission tomography combined with (low-dose) computed tomography (FDG PET/CT) has become common practice in the diagnosis of large-artery involvement in GCA [4].

One of the key issues in large artery FDG PET/CT image interpretation is the distinction between vasculitis and atherosclerosis. Atherosclerotic plaques in the wall of large arteries may show significant FDG uptake, a characteristic that is used mainly in research settings to assess the degree of plaque inflammation [5, 6]. Objective image evaluation criteria to help distinguish GCA and atherosclerosis on FDG PET/CT are lacking. Until now, several image assessment criteria have been proposed in the literature without evaluating their practical applicability. The currently used criteria are based mainly on expert opinion, and some have been evaluated merely for their ability to predict the likelihood of GCA. However, no criteria have formally been assessed in terms of their potential to distinguish vasculitis from atherosclerosis [7, 8].

The aim of our study was to perform agreement and reliability analyses of several proposed FDG PET/CT image assessment criteria. The criteria that perform well were further analysed, individually and in combination with each other, for their accuracy in distinguishing GCA from atherosclerosis. Using these criteria, a scoring system that allows for objective assessment of the likelihood of GCA was constructed. In addition, the ‘gestalt’ (overall subjective impression) conclusions drawn by different observers were compared before and after detailed image assessment. We hypothesized that a standardized scoring system of large-artery FDG PET/CT images could be at least equally good, if not better, in distinguishing vasculitis from atherosclerosis compared to ‘gestalt’ image assessment.

## Materials and methods

The study was designed and reported in accordance with the STARD guidelines for diagnostic accuracy studies (https://www.equator-network.org/reporting-guidelines/stard/).

### Patient sample

Participants were recruited both retrospectively and prospectively from 6 participating centres. The protocol was approved by the Medical Ethics Committees of involved hospitals.

The vasculitis group contained patients 50 years and older, who were initially diagnosed with probable large-artery vasculitis based on FDG PET/CT results, which diagnosis was subsequently confirmed by temporal artery biopsy. The age limit was waived in one case to include a patient of 47 years who met all other criteria for GCA. To ensure that the GCA group would be representative of daily clinical practice, absence of atherosclerosis was not a criterion for the GCA group.

Atherosclerosis patients were recruited prospectively from a database of vascular outpatients having recently (< 2 years) undergone CT angiography for identification of possible indications for vascular reconstructive surgery. Thus, all patients in this group had severe atherosclerosis in multiple vessels, as assessed by board certified vascular surgeons. After informed consent, these patients underwent additional FDG PET/CT for inclusion in the current study.

Exclusion criteria for both groups included the use of immunosuppressive drugs (corticosteroids or any other immunosuppressor), a diagnosis of cancer and reluctance or inability to provide informed consent. Several additional exclusion criteria applied to the atherosclerosis group to prevent accidental inclusion of patients with undiagnosed vasculitis: erythrocyte sedimentation rate (ESR) > 30 mmHg, C-reactive protein (CRP) > 10 mg/l, myalgia in shoulder/pelvic regions (suggesting polymyalgia rheumatica) and any history of vasculitis or systemic autoimmune disease.

All but one of the participating centres were EARL (EANM Research Ltd) certified, assuring that the FDG PET/CT scans were made according to standardized protocols designed to assure consistent quality [9].

### Image assessment

Twelve board certified nuclear medicine physicians were recruited, with clinical experience ranging from a minimum of 5 years to several decades. All 60 FDG PET/CT image sets were evaluated by these observers independently and on separate occasions. Observers were informed about the purpose of the study and were thus aware that patients were selected for having either GCA or severe atherosclerosis, but were blinded for the clinical data during evaluation of the PET/CT images. For each patient, an image set containing non-attenuation corrected series, CT-based attenuation correction series, and low-dose CT images were made available.

Assessment criteria per artery were selected based on literature review and expert consultation among nuclear medicine specialists with at least 10 years of clinical experience. The resulting criteria included FDG uptake intensity on attenuation-corrected images, compared to mediastinal blood pool (MBP) and liver uptake, as well as FDG uptake distribution over the length of the artery (‘focal’, ‘diffuse’ or ‘both focal and diffuse’, without formal/metric criteria), circularity (fully circumferential FDG uptake; assessed in the aorta only due to diameter requirements) as well as vascular wall calcification and FDG uptake calcification co-localization [10-13].

On each CT-based attenuation correction image set, the following arteries were assessed: communal carotid, vertebral, subclavian, aorta (divided into ascending, arch, descending and abdominal segments), communal iliac and femoral until mid-femur height.

On each CT image set, the observers were asked to assess the aforementioned arteries in transverse view and count each visually distinct calcification as a singular entity. The calcifications were classified as ‘small’ if they could be traced for < 3 slides and ‘large’ if they could be traced over ≥ 3 slides. *Severe* calcification was scored if > 5 small or > 1 large calcifications. *Moderate* calcification when 4–5 small calcifications or 1 large calcification were present. *Mild* calcification was scored for up to 3 small calcifications (see Supplemental Table A1for examples) [14]. An overlay of PET with CT imaging was used to assess co-localization of calcifications with FDG uptake in the vascular wall.

**Table 1:** Overview of criteria with their scoring options

18FDG uptake intensity	FDG uptake distribution	FDG uptake circularity	Calcification**	Uptake: calcification co-localization
Lower or equal to MBP (0)	Focal (0)	No (0)	None (0)	Yes (0)
In between MBP and the liver (1)	Diffuse (1)	Yes (1)	Mild (1)	Partial (1)
Equal to the liver (2)	Both (2)		Moderate (2)	No (2)
Higher than the liver (3)			Severe (3)	

*MBP* mean blood pool

The image sets were presented to the observers in random order. Observers were asked to systematically evaluate the images, starting each image series with the same artery, and judging FDG uptake intensity compared to MBP and liver, uptake distribution and circularity, followed by degree of calcification and uptake calcification co-localization in this fixed order. An overview of the categorization of these criteria can be found in Table 1. The scores (as indicated in Table 1) awarded to the separate vascular sections were registered on a standardized form. Overall impression of the image set, both prior to the systematic assessment (*gestalt* diagnosis) and after completing detailed scoring of the five criteria (final conclusion) were also provided by the observers. Observers were allowed to view the non-attenuation corrected images if they so desired and were allowed to employ additional tools such as three-dimensional maximum intensity projection, as long as the assessments made were purely visual on the basis of attenuation-corrected images.

### Statistical analysis

To select the most useful criteria, agreement and reliability of the proposed image criteria were tested first [15, 16]. After this step, the remaining criteria were analysed for discriminatory value and combined into a diagnostic model to distinguish vasculitis from atherosclerosis. The final analysis evaluated the accuracy of the diagnostic model, expressed as overall % of correct diagnoses.

### Agreement and reliability

Agreement between observers judging the same arterial segment was tested. And two-thirds (67%) consensus among the 12 observers was set as a threshold for sufficient agreement. Of the 60 image sets, the same arterial segment had to meet this 2/3 consensus in at least 50% of patients before the criterion was considered potentially appropriate for application. All criteria were also assessed for reliability by calculating ICC, as a substitute for weighted kappa with quadratic weights [17]. ICC is interpreted similarly as kappa with the advantage that ICC can be easily calculated for more than two observers. Single measure values from intra-class correlation coefficient (ICC) calculations with observers as random factors were used [17, 18]. The ICC value was classified as *very poor* (0–0.20), *poor* (0.20–0.40), *moderate* (0.40–0.60), *good* (0.60–0.80) or *excellent* (0.80–1.00) [19].

### Diagnostic performance

After criteria were selected based on agreement and reliability, discriminatory ability was evaluated using Area-Under-the-Curve (AUC) in Receiver-Operator-Characteristics (ROC) analysis [20]. In this analysis, each individual image set observation was treated as a separate case, resulting in a total of 720 cases (12 observers times 60 image sets). The criteria that showed that good discriminatory ability were combined into a scoring system and again analysed using ROC analysis. This final multi-component system was then applied to the cases aiming to determine a cut-off value that maximizes AUC. Accuracy was then calculated using these cases, defined as percentage of correctly identified image sets, and compared to the accuracy calculated from the initial and the final conclusions by the observers.

## Results

### Patients

The study included 30 patients in each study group. The GCA group comprised 17 females and 13 males, whose mean age was 71 years (range: 47–83 years; one patient was just below 50 but had biopsy-proven GCA). The atherosclerosis group comprised 7 females and 23 males whose mean age was 68 years (range: 59–83 years). The sex distribution of both groups was thus unequal, which reflects the higher incidence of GCA in females [1]. All but one vasculitis patient showed ESR > 30 mmHg at time of diagnosis. Despite this, the patient was included because all other criteria were met, including a positive temporal artery biopsy. Eight atherosclerosis patients had a vascular prosthesis.

Maximum 18F-FDG uptake and calcification scores in the respective study groups are listed in supplemental Tables A2 And A3.

### Agreement

Results of the agreement analyses are listed in Table 2. Agreement on FDG uptake intensity with 4 categories (with 2 subcategories using MBP for uptake lower than liver) scored low. Adjusting FDG uptake intensity to 3 categories (*lower than liver, equal to liver, higher than liver*) largely resolved this problem, except for the femoral artery. Agreement was generally good for calcification (except in the carotid artery) and high for distribution, circularity and co-localization.

**Table 2:** Percentage of 2/3 consensus on judged image sets arranged by criteria for each vessel on the survey list

*Arteries*	*Uptake intensity* *(4 categories)*	*Uptake intensity* *(3 categories)*	*Uptake distribution*	*Uptake circularity*	*Calcification*	*Uptake: calcification co-localization*
carotid L/R	23%/23%	**65%/65%**	**100%/100%**	n.a	47%/47%	**98%/98%**
vertebral L/R	48%/46%	**83%/83%**	**100%/100%**	n.a	**93%/93%**	**100%/100%**
subclavian L/R	38%/38%	**68%/68%**	**97%/98%**	n.a	**57%/55%**	**100%/100%**
ascending aorta	40%	**75%**	**100%**	**100%**	**67%**	**95%**
Aortic arch	40%	**72%**	**98%**	**98%**	**67%**	**92%**
Descending aorta	43%	**75%**	**92%**	**98%**	**65%**	**87%**
Abdominal aorta	30%	**67%**	**80%**	**83%**	**82%**	**88%**
Iliac L/R	13%/13%	**52%/53%**	**97%/98%**	n.a	**73%/80%**	**98%/98%**
Fem L/R	25%/27%	28%/30%	**98%/100%**	n.a	**57%/55%**	**97%/95%**

If for at least 50% of all image sets 2/3 of observers assigned the same value (indicated in bold), we considered the criterion to be suitable for that artery. *L/R* Left and right artery

### Reliability

Table 3 lists the intra-class correlation results for the assessment criteria. For FDG uptake intensity in 3 categories, reliability was moderate to good, except for the iliofemoral region. Calcification scoring also showed mostly good reliability, but less so in the subclavian and vertebral arteries. Reliability was poor for uptake distribution, circularity and co-localization. For distribution, circularity and co-localization the scoring showed a skewed distribution with most cases in a single or few categories, explaining the low reliabilities.

**Table 3:** ICC analysis by criteria for each vessel

*Arteries*	*Uptake 3 intensity* *(3 categories)*	*Uptake distribution*	*Uptake circularity*	*Calcification*	*Uptake: calcification* *Co-localization*
Carotid L/R	**0.60/0.61**	0.01/0.01	n.a	**0.62/0.62**	0.06/0.04
Vertebral L/R	0.59/0.58	0.01/-0.01	n.a	0.37/0.25	0.11/0.16
Subclavian L/R	**0.78/0.78**	0.10/0.08	n.a	0.47/0.45	0.04/0.01
Ascending aorta	**0.76**	0.04	0.04	**0.65**	0.19
Aortic arch	**0.76**	0.03	0.03	**0.74**	0.09
Descending aorta	**0.76**	0.08	0.06	**0.77**	0.14
Abdominal aorta	**0.65**	0.04	0.04	**0.71**	0.08
Iliac L/R	0.40/0.40	0.02/0.01	n.a	**0.80/0.78**	0.03/0.00
Fem L/R	0.38/0.39	0.05/0.02	n.a	**0.69/0.67**	0.04/0.05

Good (ICC 0.60–0.80) or higher reliability has been presented in bold. *L/R* Left and right artery

### ROC analysis

**Fig. 1:**
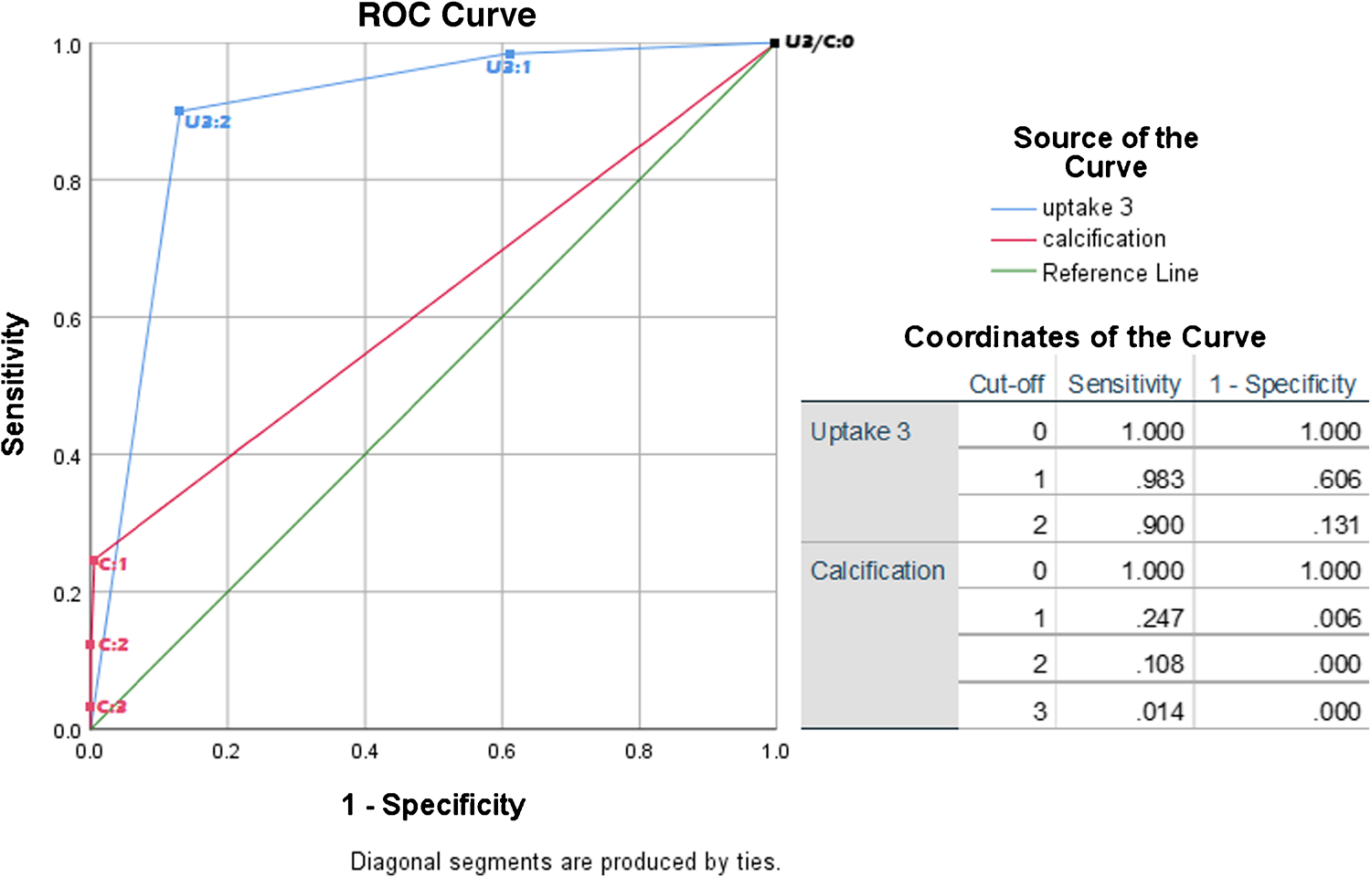
ROC curve of FDG uptake intensity in 3 categories (‘uptake 3’) and calcification. U3 = uptake 3, C = calcification. Followed by cut-off points behind semicolons

ROC analysis of uptake intensity with 3 categories and calcification (Fig. 1) revealed FDG uptake intensity to be the only discriminating criterion, with an AUC of 0.90 (95% CI 0.87–0.92). Calcification showed no relevant discrimination ability by itself, with an AUC of 0.62 (95% CI 0.58–0.66). However, as absence of calcification only occurred in GCA, calcification was combined with uptake intensity into a 2-component, 6-tiered scoring system to assess if calcification would add to the discriminative ability of uptake intensity. For this purpose, calcification was simplified to ‘present’ or ‘absent’. Combining the two criteria resulted in a 6-tiered categorization ranging from < Liver uptake + calcification, representing maximum likelihood of atherosclerosis, to > Liver uptake + no calcification, representing definite presence of vasculitis (Table 4, upper section).

**Table 4:** Overview of cases according to 6- and 4-tiered method

	Combined score	Vasculitis	Atherosclerosis	Total
**6 Tiers**				
0	< Liver + Calc	5	132	137
1	< Liver + No calc	1	10	11
2	= Liver + Calc	28	166	194
3	= Liver + No calc	2	5	7
4	> Liver + Calc	284	47	331
5	> Liver + No calc	40	0	40
	Total	360	360	720
**4 tiers**				
0	≤ Liver + Calc	33	298	331
1	≤ Liver + No calc	3	15	18
2	> Liver + Calc	284	47	331
3	> Liver + No calc	40	0	40

**Fig. 2:**
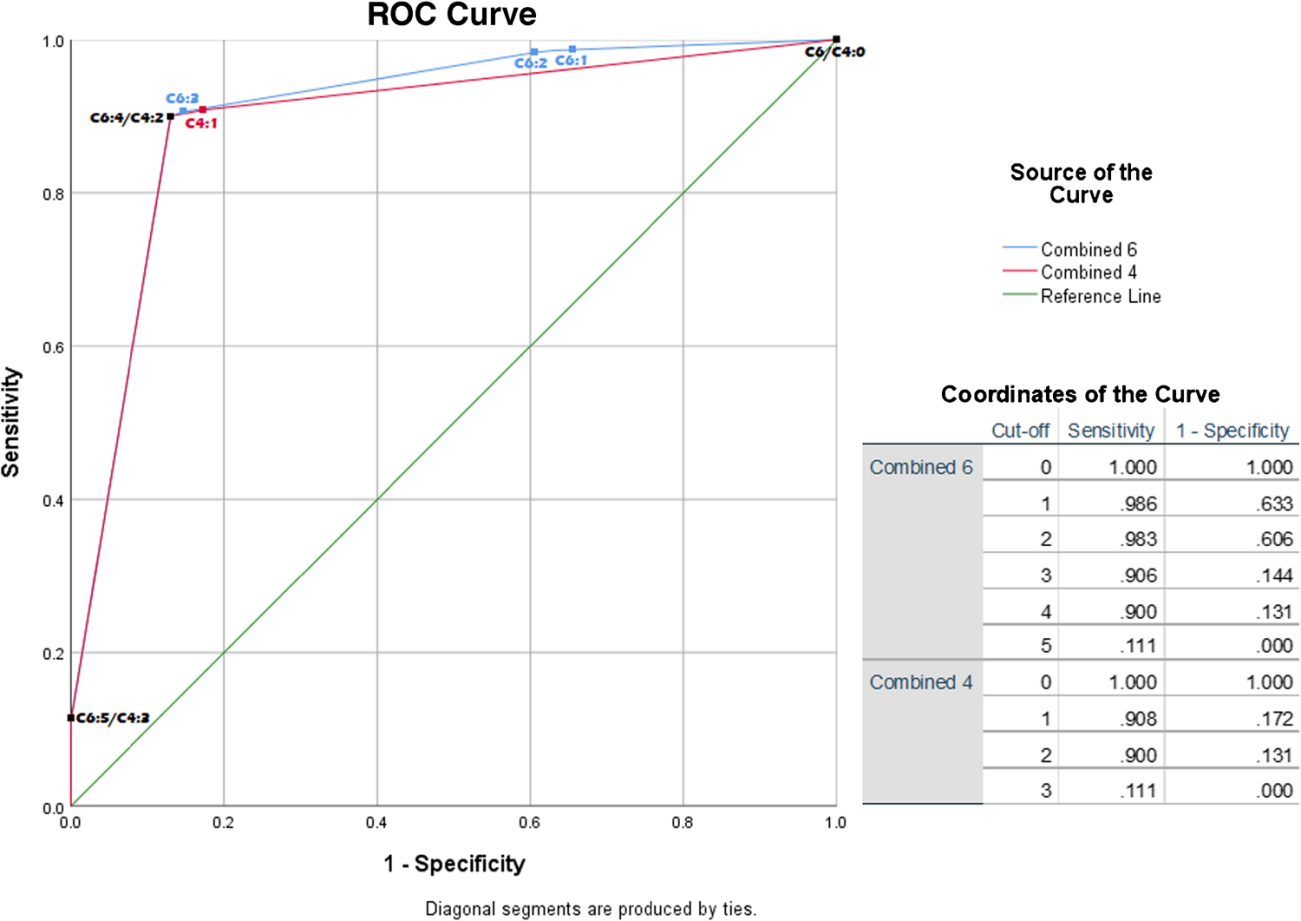
ROC curve of combined 6-component (C6) and 4-component (C4) criterion. Coordinates of the curve show cut-off values denoted as belonging to C6 or C4

Whilst the 6-tiered scoring method had similar discriminatory ability (AUC 0.91; 95% CI 0.88–0.93; Fig. 2) to uptake intensity alone, it did allow for more efficient sorting of cases. For example, the highest score in the 6-component method (uptake > liver and no calcifications) denoted certain vasculitis, whilst based on high vascular uptake alone, several atherosclerosis cases would still have been mistaken for vasculitis (Table 4, top section). Using 6 tiers did however create several small groups in some of the components. To see if these could be eliminated without affecting the AUC, the combined scoring criteria condensed into 4 tiers by merging uptake < liver and uptake = liver was also analysed (Table 4, lower section). The ROC analysis of the 4-component combined method showed an AUC that was comparable to the 6-tier method (0.8; 95%CI 0.87–0.92; Fig. 2).

**Fig. 3:**
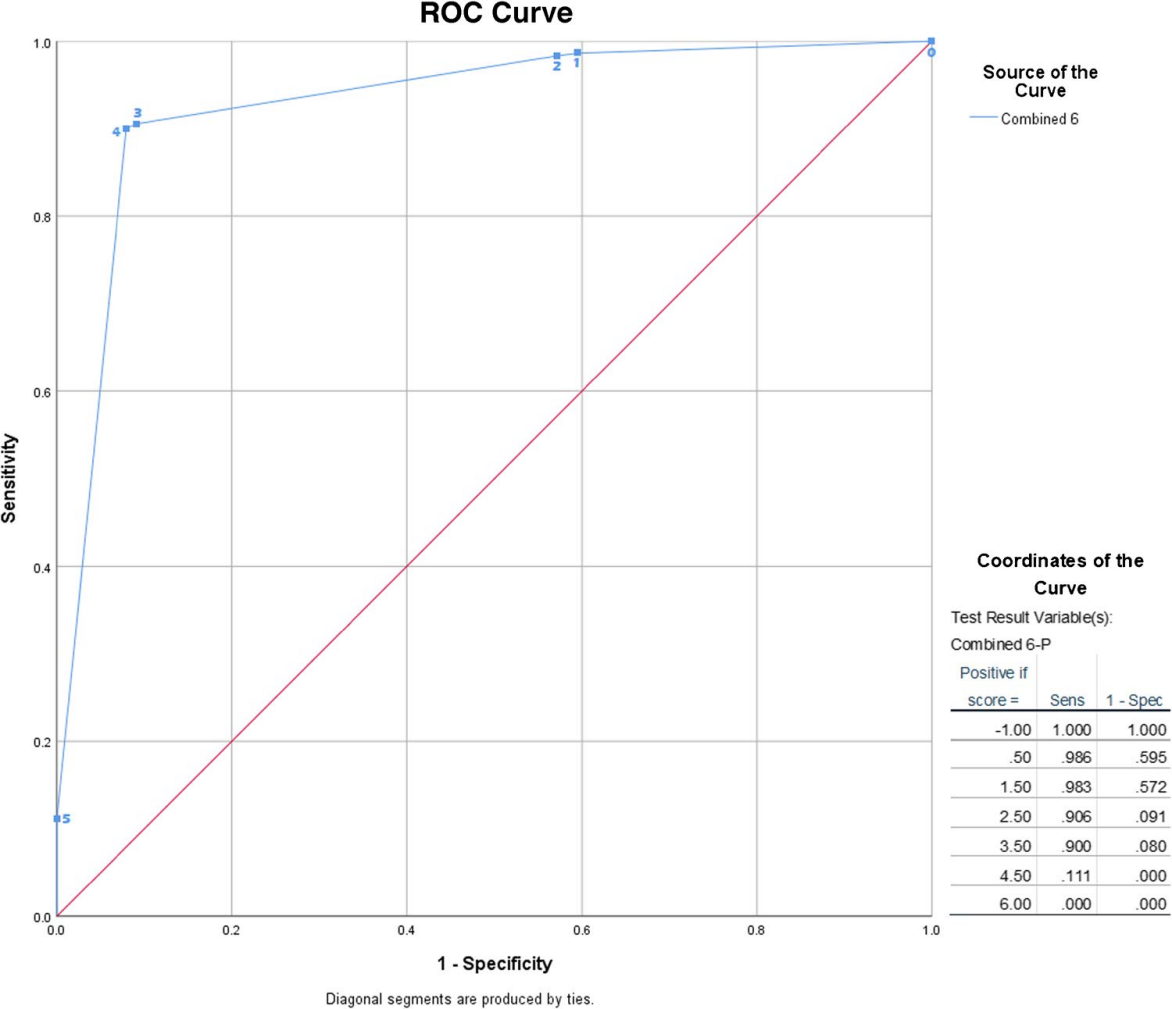
ROC curve of 6-component method without prosthesis cases

The presence of patients with vascular prostheses may have influenced the results. To evaluate this, 8 image sets with vascular prostheses were removed from the dataset. The censure of cases with prostheses resulted in an increase in AUC to 0.93 (95%CI 0.91–0.95; Fig. 3) for the 6-tiered method. This increase, albeit modest, suggested that it may be beneficial to eliminate arteries containing vascular prostheses from scoring. With the cut-off value set at tier 4, over half of patients risking misclassification based on uptake > liver were eliminated by removing prostheses, from 47 to 21 patients (Table 5). Table 6 summarizes the discriminatory value of all assessed scoring methods.

**Table 5:** Overview of 6-component method with prostheses removed

Score	Combined score	Vasculitis	Atherosclerosis
0	< Liver + Calc	5	107
1	< Liver + No calc	1	6
2	= Liver + Calc	28	127
3	= Liver + No calc	2	3
4	> Liver + Calc	284	21
5	> Liver + No calc	40	0
	Total	360	264

**Table 6:** AUC of all formally assessed scoring systems

	AUC	95% CI
Uptake only, in 3 tiers	0.90	0.87–0.92
Calcification only	0.62	0.58–0.66
4-tiered, uptake + calcification	0.89	0.87–0.92
6-tiered, uptake + calcification	0.91	0.88–0.93
6-tiered, prosthetics removed	0.93	0.91–0.95

### Accuracy

The observers’ initial (‘gestalt’) conclusion was 88.1% accurate. The accuracy of final qualitative conclusion after step-by-step visual confrontation with the scoring items was 92.8%. Accuracy of the 6-tiered method, with cut-off of 4, was 88.5%, thus similar to initial ‘gestalt’ but slightly lower than the assessors’ overall accuracy after detailed image examination. Accuracy of the 4-tier method was virtually identical to the 6-tier method. Observers adjusted their initial gestalt assessment in 36 cases, 35 of which were correct. Accuracy also increased for observers after step-by-step assessment of the images (Table 7, for 2 × 2 tables: see Supplemental Table A4).

**Table 7:** diagnostic accuracy of binary diagnoses

	Accuracy	Accuracy 95%CI
Gestalt conclusion	88.1%	95%CI 85.5–90.3%
Final conclusion	92.8%	95%CI 90.6–94.6%
6-Tier method (< 4 versus ≥ 4)	88.5%	95%CI 85.9–90.7%
4-Tier method (< 2 versus ≥ 2)	88,4%	95%CI 85.8–90.6%

Accuracy of observers increased to 93.1% (2 × 2 table: see Supplemental Table A5) for post-examination conclusions when censoring image sets containing vascular prostheses. The accuracy of the individual observers ranged from 80.8% to 98.1% (see supplemental Table A6 in appendix for individual observer accuracy).

## Discussion

The main finding of our study is that a standardised image scoring system, based on FDG intensity and calcification, for distinguishing large-artery vasculitis from atherosclerosis using 18F-FDG-PET/CT had excellent discriminatory capacity.

Agreement and reliability regarding FDG uptake intensity assessment seems to decrease for more caudally located arteries. A possible explanation for this observation could be the relatively high, but often irregular uptake in these arteries, which may be age related [21]. Based on our data, we caution against over-relying on uptake in the iliac and femoral arteries.

In clinical practice, FDG PET-CT images are predominantly evaluated by Nuclear Medicine physicians by visual assessment. Although semi-quantitative measurements like SUV-mean or SUV-max are sometimes obtained, there are no generally accepted cut-off values for daily practice, which is the domain the study was designed for. Obviously, use of liver as reference has intrinsic drawbacks. For example, the condition of the liver in relatively old patients may play a role. Steatosis and other liver conditions decrease 18F-FDG uptake in the liver resulting in a lower reference uptake [22]. Prior to switching to uptake intensity with 3 components, mean blood pool for reference uptake, which too is variable, was also used [23]. Requesting observers to use two uptake reference areas and four categories lowered agreement significantly (Table 2).

For the criteria distribution and circularity, the majority of vessels were scored as showing diffuse circular uptake. Finding focal and non-circular uptake as one would expect in atherosclerosis seems rare.

Several issues when assessing calcification were noted. Detailed grading of calcifications in 4 components seems to have no added diagnostic value compared to simple presence or absence of calcification. High-dose contrast CT imaging provides more detail and may improve accurate calcification scoring, but has the obvious drawbacks of radiation exposure and requiring intravenous contrast fluid administration [24]. Merely noting the presence or absence of calcification also resolves the lack of agreement and reliability in the carotid, vertebral and subclavian arteries.

Finally, co-localization proved to be an impractical and inaccurate criterion. The scoring of co-localization was prone to error and was most often revised by observers during scoring.

Analysis using ROC statistics suggests only uptake intensity as a useful criterion. Although including calcification does not increase AUC, it does allow for easier identification of patients with a certain diagnosis of vasculitis and is therefore preferred over just including uptake intensity.

The initial (gestalt) and final conclusions of observers showed a high degree of accuracy in distinguishing vasculitis from atherosclerosis. There was however a wide difference in the performance of the individual observers. The amount of time required by an observer to score a single case also varied widely, ranging from between 10 and 60 min. Reducing it to only two criteria significantly speeds up the process, with a conclusion being reached in less than 10 min in most cases.

One benefit of the 2-component, multitier methodology is improvement of observer accuracy, perhaps particularly for relatively inexperienced physicians. It also allows observers to categorize uncertain cases for more detailed examination. Furthermore, it may also be applied as a training tool to guide less experienced physicians in recognizing the key signs of vasculitis on FDG PET/CT.

### Strength and limitations

This study is the first to objectify criteria proposed in literature for diagnosing vasculitis on FDG PET/CT imaging. It is also the first effort to then rework these criteria into a standardised diagnostic methodology. The case control design of the study encompassed the inclusion of 50% vasculitis cases. This will deviate from prevalence within a clinical referral cohort. Depending on the reason for referral, vasculitis prevalence may fluctuate from 45% in patients suspected of GCA, to 60% in patients with persistent polymyalgia rheumatica [25, 26]. Furthermore, selective inclusion of temporal artery biopsy-proven vasculitis cases may have attributed to higher-than-average accuracy compared to a clinical setting. For example, large-artery involvement may be different in those with and without cranial involvement. Similarly, severe atherosclerotic patients were included, as these represent the main diagnostic challenge when large-artery FDG uptake is observed [5, 6]. In a clinical setting, milder degrees of atherosclerosis may affect the accuracy of standardised scoring. Clearly, if GCA patient without any degree of atherosclerosis would have been included, our study would have had no value, because GCA patients would not have been representative of the average western population around 70 years of age.

In this study, our proposed scoring method was constructed post hoc. To further examine the diagnostic performance of the scoring system, prospective testing is needed. Such prospective studies will be performed on newer PET-CT scanner equipment with improved specifications, for example, in terms of sensitivity and partial volume correction. With continuously improving equipment, any previously developed scoring system should be re-assessed for the potential of refinement. In addition, artificial intelligence-based methods may improve the future diagnostic accuracy of PET-CT.

## Conclusion

A standardised objective assessment of arterial wall FDG uptake intensity, preferably combined with assessment of arterial calcifications into a 6-tiered scoring method, showed an excellent accuracy in distinguishing large artery vasculitis from atherosclerosis.

## Supplementary Information

Below is the link to the electronic supplementary material.

Supplementary file1 (DOCX 11536 KB)

## Data Availability

The datasets generated during and/or analysed during the current study are available from the corresponding author on request.

## References

[1] Lazarewicz K, Watson P (2019). Giant cell arteritis. BMJ.

[2] Davies CG, May DJ (2011). The role of temporal artery biopsies in giant cell arteritis. Ann R Coll Surg Engl.

[3] Ing EB, Wang DN, Kirubarajan A, Benard-Seguin E, Ma J, Farmer JP, Belliveau MJ, Sholohov G, Torun N (2018). Systematic review of the yield of temporal artery biopsy for suspected giant cell arteritis. Neuroophthalmology.

[4] Hellmich B, Agueda A, Monti S, Buttgereit F, de Boysson H, Brouwer E, Cassie R, Cid MC, Dasgupta B, Dejaco C, Hatemi G, Hollinger N, Mahr A, Mollan SP, Salvarani C, Sivakumar R, Tian X, Tomasson G, Turesson C, Schmidt W, Viliger PM, Watts R, Toung C, Luqmani RA (2020). 2018 update of the EULAR recommendations for the management of large-vessel vasculitis. Ann Rheum Dis.

[5] K. Johnsrud, K. Skagen, T. Seierstad, M. Skjelland, D. Russell, M.E. Revheim(18)F-FDG PET/CT for the quantification of inflammation in large carotid artery plaques. J Nucl Cardiol 2019;**26**:883–893 10.1007/s12350-017-1121-7 PMC6517604 29209949

[6] Rudd JHF, Myers KS, Bansilal S, Machac J, Pinto CA, Rafique A, Hargeaves R, Farkouh M, Fuser V, Fayad ZA (2008). Atherosclerosis inflammation imaging with 18F-FDG PET: carotid, iliac, and femoral uptake reproducibility, quantification methods, and recommendations. J Nucl Med.

[7] Lensen KDF, Comans EF, Voskuyl AE, van der Laken CJ, Brouwer E, Zwijnenburg AT, Pereira Arias Bouda LM, Glaudemans AW, Slart RH, Smulders YM. Large-vessel vasculitis: interobserver agreement and diagnostic accuracy of 18F-FDG-PET/CT. BioMed Research Int. 2015:904692 10.1155/2015/914692 PMC4324480 25695092

[8] Stellingwerff MD, Bouwer E, Lensen KDF, Rutgers A, Arends S, van der Geest KSM, Glaudemans AW, Slart RHl. Different scoring methods of FDG PET/CT in giant cell arteritis: need for standardization. Med. 2015;**94**:e1542 10.1097/MD.0000000000001542 PMC4635818 26376404

[9] Boellaard R, Oyen WJ, Hoekstra CJ, Hoekstra OS, Visser EP, Willemsen AT, Arends B, Verzijlbergen FJ, Zijlstra J, Paans AM, Comans EJ, Pruim J (2008). The Netherlands protocol for standardisation and quantification of FDG whole body PET studies in multi-centre trials. Eur J Nucl Med Mol Imaging.

[10] Belhocine, Blockmans D, Hustinx R, Vandevivere J, Mortelmans L. Imaging of large vessel vasculitis with (18)FDG PET: illusion or reality? A critical review of the literature data. Eur J Nucl Med Mol Imag. 2003;**30**:1305–1313. 10.1007/s00259-003-1209-y 12811529

[11] Puppo, Massollo M, Paparo F, Camellino D, Piccardo A, Shoushtari Zadeh Naseri M, Villavecchia G, Rollandi GA, Cimmino MA. Giant cell arteritis: a systematic review of the qualitative and semiquantitative methods to assess vasculitis with 18F-fluorodeoxyglucose positron emission tomography. BioMed Res Int. 2014;**2014**:574248 10.1155/2014/574248 PMC4165737 25254211

[12] Balink H, Bennink RJ, van Eck-Smit BL, Verberne HJ (2014). The role of 18F-FDG PET/CT in large-vessel vasculitis: appropriateness of current classification criteria?. Biomed Res Int.

[13] Slart RHJA, Writing group; Reviewer group; Members of EANM Cardiovascular; Members of EANM Infection & Inflammation; Members of Committees, SNMMI Cardiovascular; Members of Council, PET Interest Group; Members of ASNC; EANM Committee Coordinator. FDG-PET/CT(A) imaging in large vessel vasculitis and polymyalgia rheumatica: joint procedural recommendation of the EANM, SNMMI, and the PET Interest Group (PIG), and endorsed by the ASNC. Eur J Nucl Med Mol Imaging 2018;**45**:1250–1269 10.1007/s00259-018-3973-8 PMC5954002 29637252

[14] Wintermark M, Jawadi SS, Rapp JH, Tihan T, Tong E, Glidden DV, Abedin S, Schaeffer S, Acevedo-Bolton G, Boudignon B, Orwoll B, Pan X, Saloner D (2008). High-resolution CT imaging of carotid artery atherosclerotic plaques. Am J Neuroradiol.

[15] de Vet HCW, Dikmans RE, Eekhout I (2017). Specific agreement on dichotomous outcomes can be calculated for more than two raters. J Clin Epidemiol.

[16] de Vet HCW, Mullender MG, Eekhout I (2018). Specific agreement on ordinal and multiple nominal outcomes can be calculated for more than two raters. J Clin Epidemiol.

[17] Fleiss JL, Cohen J (1973). The equivalence of weighted kappa and the intraclass correlation coefficient as measures of reliability. Educat Psychol Meas.

[18] Landis JR, Koch GG (1977). The measurement of observer agreement for categorical data. Biometrics.

[19] Trizano-Hermosilla I, Alvarado JM (2016). Best alternatives to Cronbach’s alpha reliability in realistic conditions: congeneric and asymmetrical measurements. Front Psychol.

[20] Linden A (2006). Measuring diagnostic and predictive accuracy in disease management: an introduction to receiver operating characteristic (ROC) analysis. J Eval Clin Prac.

[21] Bural GG, Torigian D, Rubello D, Alavi A (2016). Atherosclerotic 18F-FDG and MDP uptake in femoral arteries, changes with age. Nucl Med Commun.

[22] Salomon T, Nganoa C, Gac AC, Fruchart C, Damaj G, Lasnon C (2017). Assessment of alteration in liver 18F–FDG uptake due to steatosis in lymphoma patients and its impact on the Deauville score. Eur J Nucl Med Mol Imaging.

[23] Boktor RR, Walker G, Stacey R, Gledhill S, Pitman AG (2013). Reference range for intrapatient variability in blood-pool and liver SUV for 18F-FDG PET. J Nucl Med.

[24] Kalra MK, Becker HC, Enterline DS, Lowry CR, Molvin LZ, Singh R, Rybicki FJ (2019). Contrast administration in CT: a patient-centric approach. J Am Coll Radiol.

[25] Taimen K, Salomaki SP, Hohenthal U, Kajander MM, Seppanen M, Nuutila P, Palomaki A, Roivanen A, Pirila L, Kemppainen J. The clinical impact of using ^18^F-FDG-PET/CT in the diagnosis of suspected vasculitis: the effect of dose and timing of glucocorticoid treatment. Contrast Media Mol Imaging 2019:9157637 10.1155/2019/9157637 PMC6735179 31531005

[26] Prieto-Peña D, Martínez-Rodríguez I, Loricera J, Banzo I, Calderon-Goerke M, Calo-Rio V, Gonzales-Vela C, Corrales A, Castaneda S, Blanco R, Hernandez JL, Gonzales-Gay MA*.* Predictors of positive (18)F-FDG PET/CT-scan for large vessel vasculitis in patients with persistent polymyalgia rheumatic Semin Arthritis Rheum 2019;**48**:720–727 10.1016/j.semarthrit.2018.05.007 29903537

