# Supplementary material for: Development of a simple standardized scoring system for assessing large vessel vasculitis by 18F-FDG PET-CT and differentiation from atherosclerosis
Source: Eur J Nucl Med Mol Imaging. 2023 Apr 28;50(9):2647–55. doi: 10.1007/s00259-023-06220-5 (PMC10317865; doi:10.1007/s00259-023-06220-5)
Supplement: Supplementary file 1 — Supplementary file1 (DOCX 11536 KB) [file 259_2023_6220_MOESM1_ESM.docx]

**Supplementary Information:**

| **Table A1: Definition of calcifications categories with visual examples of atherosclerosis in the abdominal aorta** | | | | |
| --- | --- | --- | --- | --- |
| Categories | | Definition | Coronal view | Transverse view |
| 0 | Severe | **>5** calcifications or **≥2** calcifications extending for **≥3** slices | 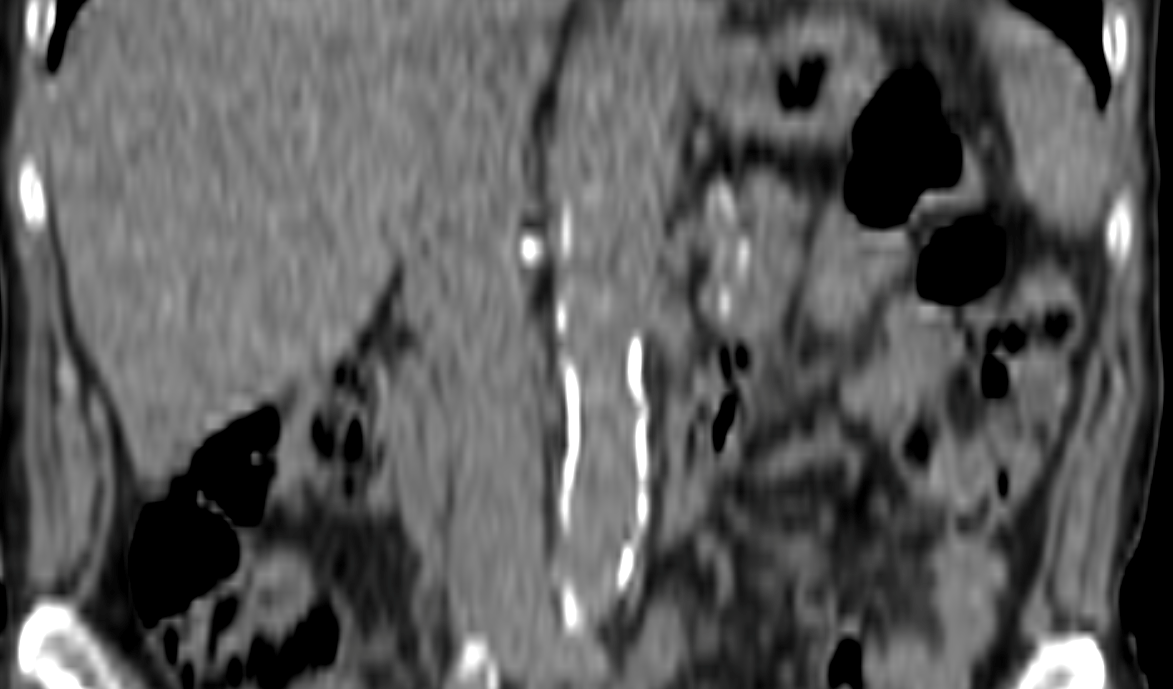 | 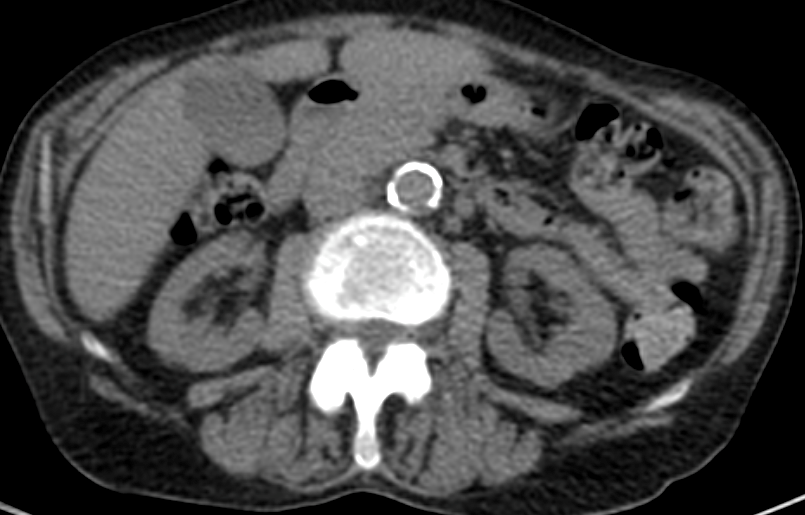 |
| 1 | Moderate | **4-5** calcifications or **1** calcification extending for **≥3 slices** | 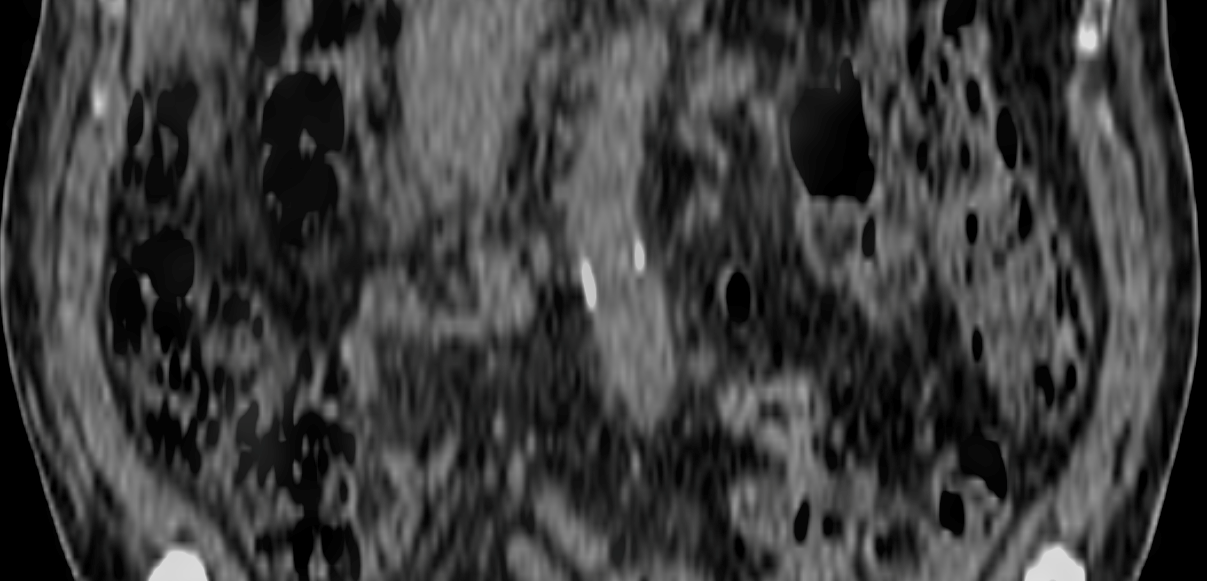 | 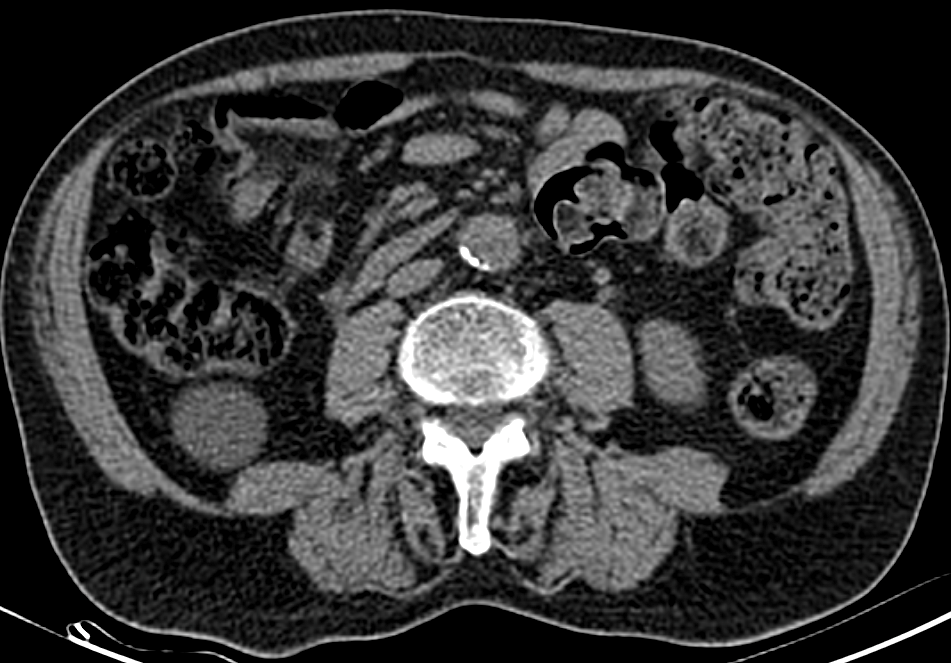 |
| 2 | Mild | **≤3** calcifications | 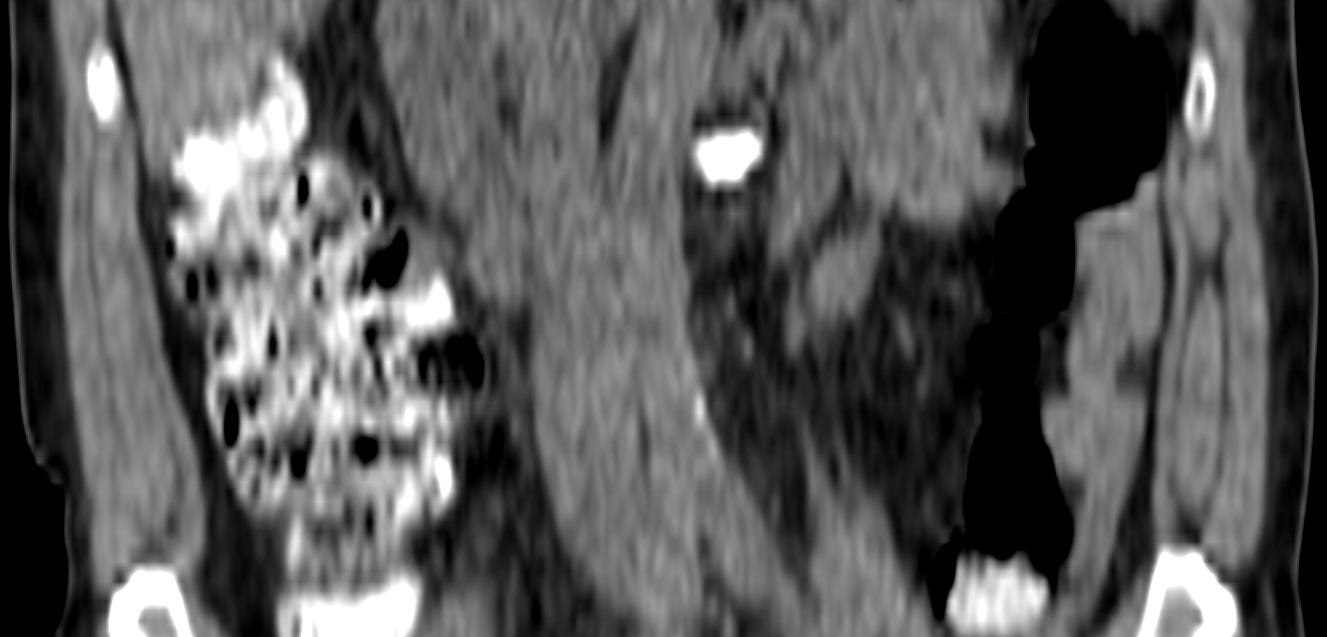 | 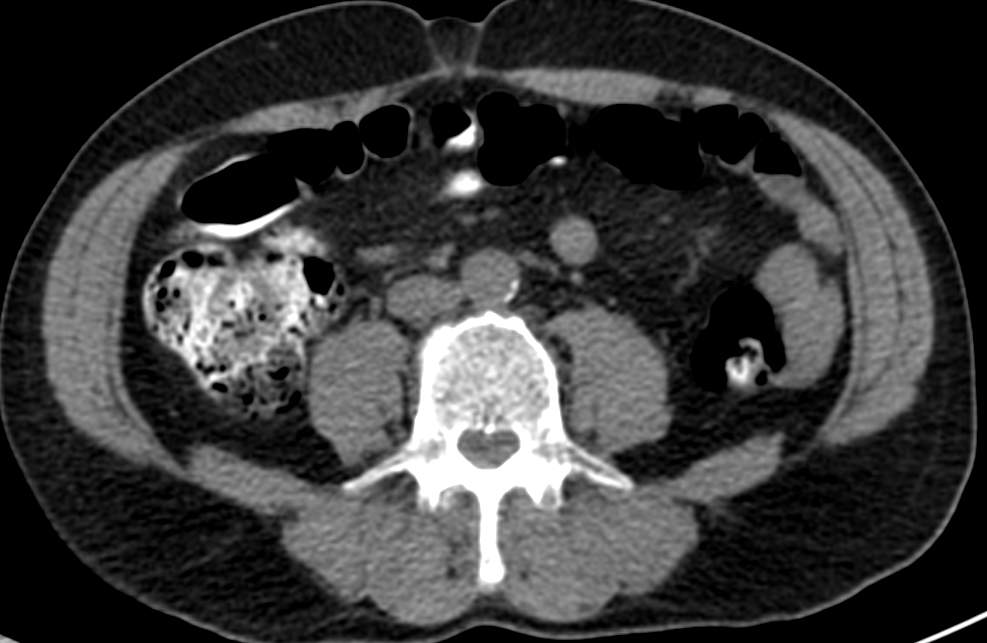 |
| 3 | None | No visible calcifications | 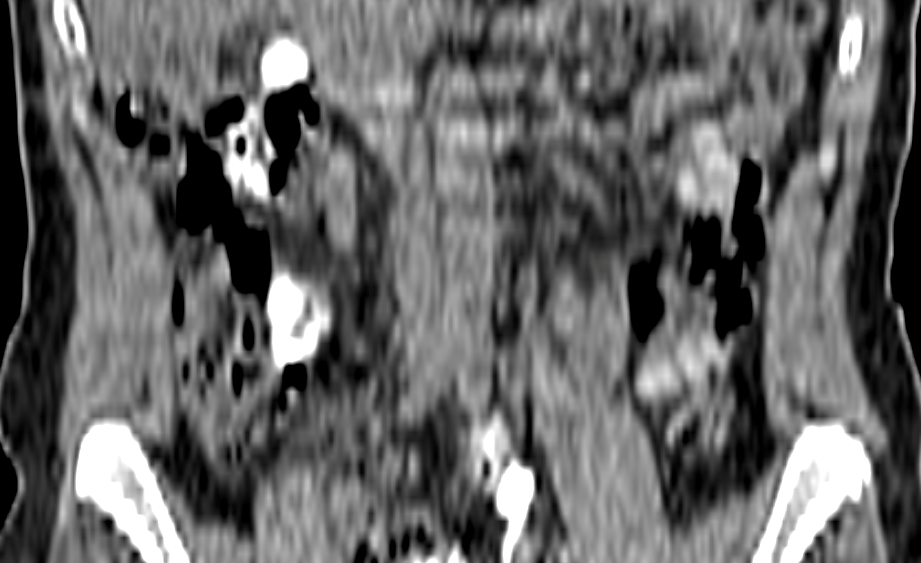 | 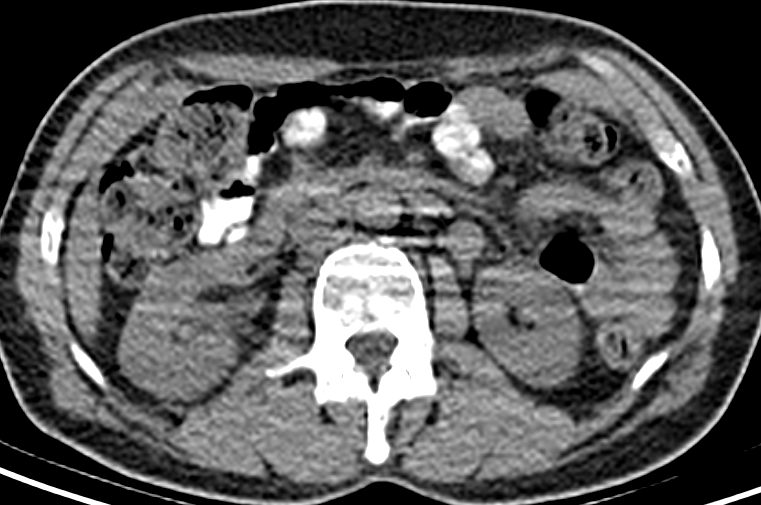 |

| **Table A2: Highest uptake score assigned to any vessel per case** | | |
| --- | --- | --- |
| Uptake | Vasculitis | Atherosclerosis |
| ≤ MBP | 1 | 55 |
| MBP - Liver | 5 | 87 |
| ≈ Liver | 30 | 171 |
| > Liver | 324 | 47 |
| Total | 360 | 360 |

| **Table A3: Most severe calcification score assigned to any vessel per case** | | |
| --- | --- | --- |
| Calcification | Vasculitis | Atherosclerosis |
| Severe | 271 | 358 |
| Moderate | 50 | 2 |
| Mild | 34 | 0 |
| None | 5 | 0 |
| Total | 360 | 360 |

| **Table A4: Conclusions drawn by observers prior and after taking the survey.** | | | | |
| --- | --- | --- | --- | --- |
|  | Condition | | | |
|  | x | Vasculitis | Atherosclerosis | Total |
| Initial ‘gestalt’ conclusion | Vasculitis | 309 | 30 | 339 |
|  | Atherosclerosis | 43 | 325 | 368 |
|  | Indiscernible | 8 | 5 | 13 |
| Total |  | 360 | 360 | 720 |
| Correct percentage | | 85,8% | 90,3% | 88,1% |
|  | Condition | | | |
|  | x | Vasculitis | Atherosclerosis | Total |
| Final conclusion | Vasculitis | 332 | 24 | 356 |
|  | Atherosclerosis | 28 | 336 | 364 |
|  | Indiscernible | 0 | 0 | 0 |
| Total |  | 360 | 360 | 720 |
| Correct percentage | | 92,2% | 93,3% | 92,8% |
| Scans belonging to vasculitis/atherosclerosis patients that were judged as indifferent are noted separately. | | | | |

| **Table A5: Conclusions drawn after taking the survey with prosthetic cases removed.** | | | | |
| --- | --- | --- | --- | --- |
|  | Condition | | | |
|  | x | Vasculitis | Atherosclerosis | Total |
| Final conclusion | Vasculitis | 332 | 15 | 337 |
|  | Atherosclerosis | 28 | 249 | 277 |
|  | Indiscernible | 0 | 0 | 0 |
| Total |  | 360 | 264 | 624 |
| Correct percentage | | 92.22% | 94.32% | 93.11% |

| **Table A6: Accuracy of each observer for final conclusions with censored prostheses** | | | | | |
| --- | --- | --- | --- | --- | --- |
| **Observer 1** | **Observer 2** | **Observer 3** | **Observer 4** | **Observer 5** | **Observer 6** |
| 88.46%  (76.56-95.65%) | 94.23%  (84.05-98.79%) | 98.08%  (89.74-99.95%) | 94.23% (84.05-98.79%) | 92.31%  (81.46-97.86%) | 96.15% (86.79-99.53%) |
| **Observer 7** | **Observer 8** | **Observer 9** | **Observer 10** | **Observer 11** | **Observer 12** |
| 98.08%  (89.74-99.95%) | 80.77%  (67.47-90.37%) | 98.08%  (89.74-99.95%) | 98.08%  (89.74-99.95%) | 92.31%  (81.46-97.86%) | 86.54%  (74.21-94.41%) |
